# RK-33 Is a Broad-Spectrum Antiviral Agent That Targets DEAD-Box RNA Helicase DDX3X

**DOI:** 10.3390/cells9010170

**Published:** 2020-01-09

**Authors:** Sundy N. Y. Yang, Sarah C. Atkinson, Michelle D. Audsley, Steven M. Heaton, David A. Jans, Natalie A. Borg

**Affiliations:** 1 Nuclear Signaling Laboratory, Monash Biomedicine Discovery Institute and Department of Biochemistry and Molecular Biology, Monash University, Clayton, VIC 3800, Australia; sundyniyenyang@gmail.com; 2 Infection & Immunity Program, Monash Biomedicine Discovery Institute and Department of Biochemistry and Molecular Biology, Monash University, Clayton, VIC 3800, Australia; sarah.atkinson@monash.edu (S.C.A.); michelle.audsley@monash.edu (M.D.A.); steven.heaton@monash.edu (S.M.H.)

**Keywords:** DDX3X, DEAD-box helicase, RK-33, viral infection, small molecule inhibitor

## Abstract

Viral disease is one of the greatest burdens for human health worldwide, with an urgent need for efficacious antiviral strategies. While antiviral drugs are available, in many cases, they are prone to the development of drug resistance. A way to overcome drug resistance associated with common antiviral therapies is to develop antivirals targeting host cellular co-factors critical to viral replication, such as DEAD-box helicase 3 X-linked (DDX3X), which plays key roles in RNA metabolism and the antiviral response. Here, we use biochemical/biophysical approaches and infectious assays to show for the first time that the small molecule RK-33 has broad-spectrum antiviral action by inhibiting the enzymatic activities of DDX3X. Importantly, we show that RK-33 is efficacious at low micromolar concentrations in limiting infection by human parainfluenza virus type 3 (hPIV-3), respiratory syncytial virus (RSV), dengue virus (DENV), Zika virus (ZIKV) or West Nile virus (WNV)—for all of which, no Food and Drug Administration (FDA)-approved therapeutic is widely available. These findings establish for the first time that RK-33 is a broad-spectrum antiviral agent that blocks DDX3X’s catalytic activities in vitro and limits viral replication in cells.

## 1. Introduction

Viral infections pose a risk to human health worldwide, with an urgent need for efficacious antiviral strategies. The relatively few antivirals in use typically function by targeting a specific viral protein critical to viral replication, and hence are only effective against a single pathogen. Although potentially effective in the short-term, their use can lead to selection for drug resistant strains that threaten the long-term effectiveness of the treatment [1]. To mitigate the risk of developing drug-resistant strains, one strategy is to instead target host cellular cofactors critical to viral replication.

DEAD-box helicase 3 X-linked (DDX3X) is a cellular adenosine 5′-triphosphate (ATP)-dependent RNA helicase that hydrolyzes ATP to unwind duplex RNA, thereby remodeling RNA/RNA–protein complexes; these catalytic functions are attributable to highly conserved sequence motifs distributed over two recombinase A (RecA)-like helicase domains [2,3]. As an ATP-dependent RNA helicase, DDX3X has roles in all aspects of RNA metabolism including transcriptional regulation, pre-mRNA splicing, messenger ribonucleoprotein particle (mRNP) assembly, and mRNA export/translation [4]. Accordingly, DDX3X is a prime host target for multiple viruses of medical significance; it is required for the replication of human immunodeficiency virus type 1 (HIV-1) [5,6], and flaviviruses such as dengue virus (DENV) and hepatitis C (HCV) [7,8]. Accordingly, DDX3X is an attractive host cellular co-factor for drug targeting.

The small molecule RK-33 (diimidazo [4,5-*d*:4′,5′-*f*]-[1,3]diazepine) is a ring-expanded nucleoside (REN) analogue [9,10] that has been suggested to target DDX3X based on the observation that biotinylated RK-33 can immunoprecipitate endogenous DDX3X in cells, and that RK-33 can inhibit helicase activity of Ded1p, the yeast orthologue of DDX3X [11]. RK-33 has been reported to effect G1 cell cycle arrest [12,13], apoptosis [11], impair Wingless-related integration site (Wnt) signaling [11,14], reduce mitochondrial translation [15], and reduce tumor proliferation/growth in xenograft mouse models of Ewing sarcoma, lung cancer and prostate cancer, with or without parallel radiation dosing [11,13,14,15,16]. RK-33 is thus of interest as a broad-spectrum anticancer agent. 

Since (1) DDX3X is an essential host co-factor for the replication of multiple viruses, (2) inhibitors of DDX3X can act as broad-spectrum antivirals [17,18] and (3) REN analogues have antiviral properties [19,20,21,22,23], we decided to test RK-33′s ability to inhibit infection by viruses belonging to the *Paramyxoviridae* and *Flaviviridae* families. Here, we validate DDX3X as a target of RK-33 for the first, time using a range of analytical ultracentrifugation, isothermal titration calorimetry and in vitro ATPase and RNA unwinding assays. Importantly, using cell-based infectious assays, we establish for the first time that RK-33 has antiviral activity against not only human parainfluenza virus type 3 (hPIV-3) and respiratory syncytial virus (RSV), but also several flaviviruses including DENV serotype 2 (DENV-2), Zika virus (ZIKV) and West Nile virus (WNV). These results highlight RK-33 as a broad-spectrum antiviral agent and support the notion that DDX3X is a viable drug target.

## 2. Materials and Methods

### 2.1. Inhibitor

RK-33 of ≥98% purity, as determined by Proton Nuclear Magnetic Resonance (^1^H-NMR), was sourced from AdooQ^®^ BioScience (Irvine, CA, USA). For in vitro experiments and cell-based infectious assays, an RK-33 stock was made up to 50 mM in 100% dimethyl sulfoxide (DMSO).

### 2.2. Protein Expression and Purification

**Figure 1:**
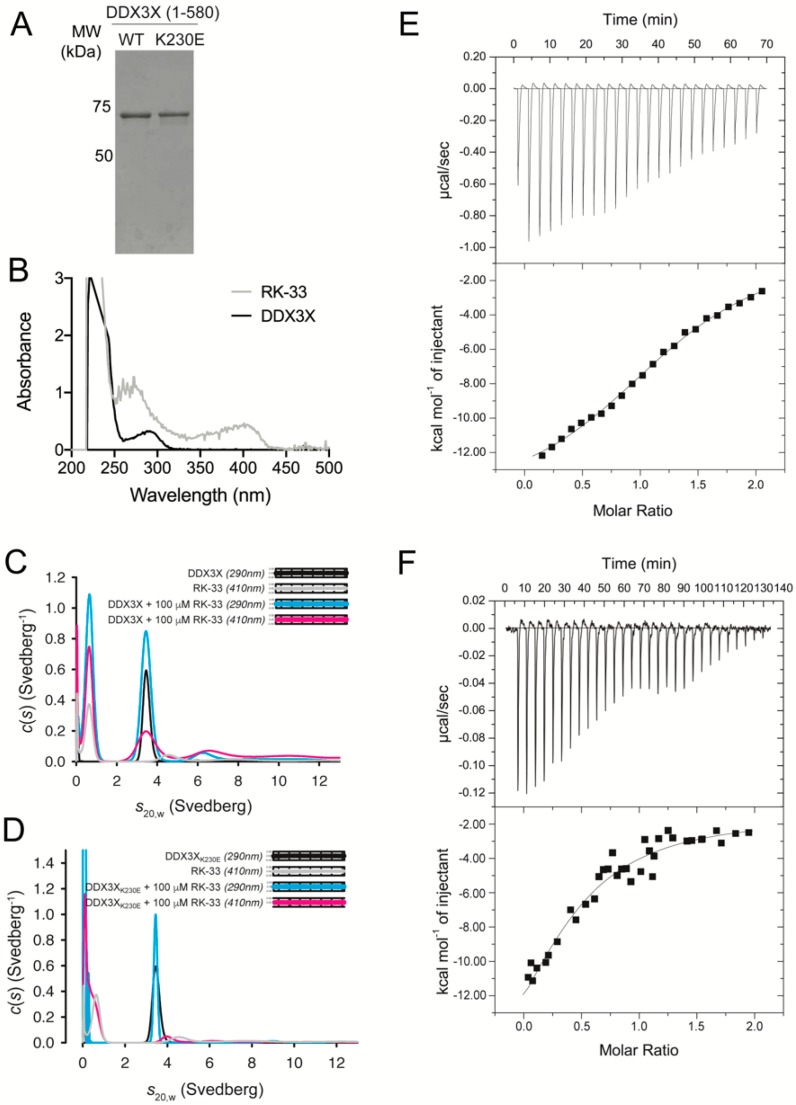
RK-33 directly binds to DEAD-box helicase 3 X-linked (DDX3X) residue K230. (**A**) Coomassie-stained SDS-PAGE of purified recombinant wild-type and K230E DDX3X. (**B**) Wavelength scans of 200–500 nm collected in the analytical ultracentrifuge on purified recombinant DDX3X alone (black) and 100 μM RK-33 (gray). Sedimentation velocity studies of RK-33 binding to (**C**) DDX3X or (**D**) DDX3X K230E. *c*(*s*) distributions from fits to data collected at 290 and 410 nm were plotted as a function of *s*_20,w_ for DDX3X in the presence of 100 μM RK-33 at 290 nm (blue) and 410 nm (pink), 100 μM RK-33 alone (410 nm, gray) and DDX3X with an equivalent % dimethyl sulfoxide (DMSO) (DDX3X alone, 290 nm, black). The residual plots are shown in insets. Data in (**C**,**D**) are each representative of two independent experiments. (**E**,**F**) Calorimetry studies of RK-33 binding to DDX3X. RK-33 at (**E**) 50 μM and (**F**) 200 μM was injected into DDX3X (17 μM) in the sample cell. Data were fit to a one-site binding model. The stoichiometry deduced from data fitting to the two experiments was 1.05 ± 0.2, with a *K*_d_ of 33 ± 2 μM and Δ*H* of −19 ± 0.3 kcal/mol. Reported values represent the average of the two experiments, with average errors of the fits to the experimental data reported by Origin 7.0.

Sequences encoding an N-terminal hexa-histidine (6His) tag upstream of human DDX3X (residues 1–580) or the catalytically inactive DDX3X mutant K230E (residues 1–580) [6] were cloned into the pCOLD expression vector (Takara Bio, Kusatsu, Japan). The 6His-DDX3X and the pCOLD-6His-DDX3X K230E mutant were expressed separately in *Escherischia coli* (*E. coli*) BL21 (DE3) cells at 16 °C following induction at OD_600nm_ = 0.6 with 1 mM isopropyl 1-thiol-β-D-galactopyranoside (IPTG) and harvested 20 h post-induction prior to resuspension in lysis buffer (20 mM Tris-Cl pH 8.0, 500 mM NaCl, 20% (*v*/*v*) glycerol, 0.5 mM TCEP, 10 mM imidazole, supplemented with 1 mg/mL lysozyme, 0.1% (*v*/*v*) Tween-20 and cOmplete protease inhibitor tablets (Roche, Basel, Switzerland)). Proteins were extracted by sonication and clarified prior to purification using Ni-NTA Superflow resin (Qiagen, Hilden, Germany). Resin was washed with lysis buffer + 20 mM imidazole and 1% (*v*/*v*) Tween-20 and eluted in buffer containing 300 mM imidazole. Eluate was then applied to a Superdex 200 16/60 gel filtration column equilibrated in 20 mM Tris-Cl pH 8.0, 500 mM NaCl, 20% (*v*/*v*) glycerol, and 0.5 mM TCEP. Protein fractions containing DDX3X were pooled and protein purity determined by SDS-PAGE (Figure 1A).

### 2.3. Analytical Ultracentrifugation

Sedimentation velocity experiments were conducted using an Optima Analytical Ultracentrifuge (Beckman Coulter, Brea, CA, USA) at a temperature of 20 °C. Protein was diluted in 20 mM Tris, pH 8.0, 150 mM NaCl, 10% (*v*/*v*) glycerol, 1 mM TCEP and where relevant, 100 μM RK-33 or an equivalent % DMSO was added prior to centrifugation. In total, 380 μL of sample and 400 μL of reference solution were loaded into a conventional double sector quartz cell and mounted in a Beckman 8-hole An-50 Ti rotor (Beckman Coulter, Brea, CA, USA). Samples were centrifuged at a rotor speed of 40,000 rpm and the data were collected continuously at multiple wavelengths (290 and 410 nm). Solvent density (1.0331 g/mL at 20 °C) and viscosity (1.385 cP at 20 °C), as well as estimates of the partial specific volume (0.7247 mL/g for DDX3X at 20 °C), were calculated using the program SEDNTERP (Durham, NH, USA) [24]. Sedimentation velocity data were fitted to a continuous size (*c*(*s*)) distribution model using the program SEDFIT (Bethesda, MD, USA) [25].

### 2.4. Isothermal Titration Calorimetry (ITC)

Measurements were made using a MicroCal VP-ITC (Malvern Panalytical, Malvern, UK) at 25 °C. For all measurements, DDX3X protein was dialyzed overnight against 20 mM Tris, pH 8.0, 150 mM NaCl, 10% (*v*/*v*) glycerol, 1 mM TCEP, and this dialysis buffer used to dilute RK-33. RK-33 (50 μM or 200 μM in 7.5% (*v*/*v*) dimethyl sulfoxide (DMSO)), or an equivalent % DMSO, was titrated into a solution containing DDX3X (17 μM). Measurements were made using 7.5 µL injections and 180 s between each injection. ITC data were analyzed with Origin software version 7.0 (OriginLab, Northampton, MA, USA).

### 2.5. RNA-Dependent ATP Hydrolysis Assay 

DDX3X-mediated ATP hydrolysis was measured using a modification of the protocol of Epling et al. [26], except that activity was measured in the presence of poly(I:C), a synthetic analogue of dsRNA (InvivoGen, San Diego, CA, USA), to stimulate ATPase activity. Reactions were performed in pentaplicate in a final volume of 50 μL in 96-microwell assay plates (Corning, Corning, NY, USA) and reagents diluted using diethylpyrocarbonate (DEPC)-treated water. Reactions consisted of ATPase reaction buffer (20 mM Tris-Cl pH 7.5, 1.5 mM DTT, 1.5 mM MgCl_2_), 200 nM DDX3X (residues 1–580) or the DDX3X K230E (residues 1–580) variant as a negative control, 0.02 mg/mL poly(I:C), with RK-33 (0–250 μM) or equivalent (2% (*v*/*v*)) DMSO, and 0.3 mM ATP (Sigma-Aldrich). Following incubation for 60 min at room temperature, 100 μL Biomol^®^ Green (Enzo Life Sciences, Farmingdale, NY, USA), a colorimetric reagent that binds to free inorganic phosphate (Pi), was added to all wells. After 15 min at room temperature, absorbance at 620 nm, which correlates with the detection of Pi, was measured using a FLUOstar Omega plate reader (BMG Labtech, Ortenberg, Germany). Phosphate standards (Enzo Life Sciences, Farmingdale, NY, USA) were serially diluted (0–40 μM) to generate a standard curve to enable quantitation of the Pi generated by DDX3X catalytic activity in the assay. 

### 2.6. RNA Unwinding Assay

RNA binding assays were performed based on the protocol of Kim and Seo [27], but with the modifications described below. The RNA substrate, based on a sequence previously shown to act as a DDX3X substrate [28], comprised annealed partially complementary single-stranded (ssRNA) molecules synthesized by Integrated DNA Technologies (IA, USA): a Cy5-labeled 15-nt RNA strand (5′-Cy5-CGUCUUUACGGUGCU-3′) and a 41-nt RNA strand, 5′-AGCACCGUAAAGACGGUAAAACAAAACAAAACAAAACAAAA-3′, where the duplex region is underlined). A 15-nt duplex with 26-nt 3′ overhang was generated by combining equimolar concentrations of the 15- and 41-nt RNAs in annealing buffer (60 mM KCl, 6 mM HEPES-pH 7.5, 0.2 mM MgCl_2_) and heating to 95 °C before slowly cooling to room temperature. Reaction mixtures (20 μL) contained 25 mM Tris-HCl, pH 7.8, 2 mM MgCl_2_, 2 mM dithiothreitol, 0.1 mg/mL BSA, 15 fmol of duplexed RNA, 0.3 mM ATP in the presence of RK-33 (0–250 μM) or equivalent (2% (*v*/*v*)) DMSO. Reactions were initiated by the addition of 300 nM DDX3X (residues 1–580) or DDX3X K230E (residues 1–580) as the negative control. After incubation at 37 °C for 30 min, reactions were stopped by the addition of 4 μL of a solution containing 0.6% SDS, 60 mM EDTA, 40% (*w*/*v)* sucrose and 0.25% bromophenol blue. As a positive control, duplex RNA was separated into monomers by heating at 95 °C for 5 min, followed by rapid cooling in ice. Samples were electrophoresed on a 15% polyacrylamide gel in 0.5x Tris-borate-EDTA (TBE). Cy5-labeled RNA was visualized by fluorescence using a Typhoon 5 (GE Healthcare, Chicago, IL, USA) and quantified using Image Studio Lite (Li-Cor, Lincoln, NE, USA). The percentage of unwinding was calculated using the formula (monomer/total) × 100, where “total” is the amount of monomer plus duplex.

### 2.7. Cell Culture and Virus Propagation 

Vero (African green monkey kidney) and baby hamster kidney (BHK)-21 cell lines were maintained in Dulbecco’s modified eagle serum (DMEM) media and C6/36 (*Aedes albopictus*) cells were maintained in basal medium eagle (BME) media supplemented with 10% heat-inactivated fetal bovine serum (FBS) at 37 °C (28 °C for C6/36 cells) in a humidified incubator supplemented with 5% CO_2_ [29]. Viral stocks of DENV-2 (New Guinea C; M29095) were propagated in C6/36 cells [30], and RSV (A2 strain) [31], hPIV-3 (GenBank accession no. AY283063), ZIKV (Asian/Cook Islands/2014) [32] and WNV (Kunjin; MRM61C strain) in Vero cells; cells at 80% confluency were infected at a multiplicity of infection (MOI) of 0.1. At 48 h, when >70% of the cells were detached, the supernatant (cell associated sample for RSV) was harvested as the virus stock. Viral titre was subsequently determined by plaque assay (see below).

### 2.8. Viral Infection and RK-33 Treatment

Vero cells were infected with DENV-2, RSV, hPIV-3, ZIKV and WNV at an MOI 1 for 2 h, after which virus was removed, and fresh medium containing 2% fetal bovine serum (FBS) containing the indicated concentration (0.01, 0.04, 0.16, 0.63, 1.25, 2.5, 10, 20, 50 μM) of RK-33 or equivalent volume of the vehicle DMSO. Culture medium was collected 22 h later and viral titres determined by plaque assay or RT-qPCR. 

### 2.9. Plaque Assay

BHK-21 (used for DENV-2) or Vero (used for RSV, hPIV-3, ZIKV and WNV) cells were seeded into 24-well plates at a density of 2 × 10^5^ cells/well and grown overnight in culture medium at 37 °C with 5% CO_2_ prior to infection. The virus inoculum was removed and replaced with semisolid overlays of 0.8% aquacide II (Calbiochem, San Diego, CA, USA) in DMEM containing 2% FBS, and the mixture was incubated at 37 °C with 5% CO_2_. After 3–4 days, the cells were fixed with neutral buffered formalin (Sigma-Aldrich, St. Louis, MO, USA) for 2 h at room temperature, rinsed with tap water, and stained with 1% crystal violet for 10 min. The stain was removed by rinsing the cells with tap water, and the viral plaques were counted visually. Dose-response curves were plotted from the plaque number (plaque forming units (pfu)/mL) versus the logarithmic value of the concentrations of RK-33. Statistical analysis was performed using GraphPad Prism 8 software. Outlier points were excluded.

### 2.10. Quantitative Reverse Transcription Polymerase Chain Reaction (qRT-PCR) 

qRT-PCR to estimate the number of viral genomes was performed as previously for RSV [31], ZIKV [32] and DENV-2 [30]. For WNV and hPIV-3 infections, supernatants from infected Vero cell cultures were extracted using the Isolate II RNA extraction kit (Bioline, London, UK), and the absolute number of RNA copies determined using TaqMan Fast Virus 1-Step Master Mix (Applied Biosystems, Foster City, CA, USA) by extrapolation from a standard curve generated from in vitro-transcribed WNV and hPIV-3 RNA. qRT-PCR conditions were 50 °C for 5 min, 95 °C for 20 s, 40 cycles of 95 °C for 3 s and 60 °C for 30 s. RT-PCR was used to detect hPIV-3 or WNV mRNA. Primers and probes used were: WNV probe: 5′-6FAM-TCACACTCTTCCGGCTGTCAATCAC-3′; WNV primers: 5′-CAAACTTTAAGGCAAGCAGGG-3′ and 5′-ATTCCTACCAATGCGTCCTC-3′. hPIV-3 probe: 5′-6FAM-CCCGGGACACCCAGTTGTGTTGCA-3′; hPIV-3 primers: 5′-CCATCTGTTGGACCAGGGAT-3′ and 5′-TGATTGCAGTCCCTCTGTGT-3′. Dose-response curves were plotted from the viral RNA copies versus the logarithmic value of the concentrations of RK-33. Statistical analysis was performed using GraphPad Prism 7 software (San Diego, CA, USA). Outlier points were excluded.

### 2.11. Cell Cytotoxicity Assay

Cell viability was determined by XTT (sodium 30-[1-[(phenylamino)-carbony]-3,4-tetrazolium]-bis(4-methoxy-6-nitro) benzene-sulfonic acid hydrate) assay as previously described [30], using XTT sodium salt (Sigma-Aldrich, St. Louis, MO, USA) and phenazine methosulfate (PMS, Sigma-Aldrich, St. Louis, MO, USA). Cells were treated the same as for the infection assay with increasing concentrations of RK-33, and XTT added 22 h later.

## 3. Results

RK-33 is a REN with anticancer properties [11,13,14,15,16] speculated to target the ATP binding site of DDX3X [11]. Since DDX3X plays a role in the replication of various viruses [5,6,7,8], we set out here for the first time to confirm RK-33′s ability to bind directly to DDX3X, inhibit its enzymatic activities, and test its ability to inhibit a range of viral infections.

### 3.1. RK-33 Binds Directly to the Active Site of DDX3X

To confirm direct binding of RK-33 to recombinant human DDX3X (Figure 1A), we exploited the spectroscopic properties of RK-33, which absorbs strongly between 330 and 430 nm (Figure 1B), in multi-wavelength analytical ultracentrifugation experiments. We collected sedimentation velocity data at 290 and 410 nm; the latter enabled the specific detection of RK-33 either alone or in complex with DDX3X, or DDX3X K230E that harbors a substitution within motif I (Walker A) and is deficient in ATP hydrolysis and RNA-unwinding [6]. At 290 nm and in the absence of RK-33, both DDX3X (Figure 1C, black) and DDX3X K230E (Figure 1D, black) sedimented as single species with sedimentation coefficients (*s*_20,w_) of 3.6S. DDX3X data collected at both 290 and 410 nm and in the presence of 100 μM RK-33 also revealed a 3.6S species, indicating RK-33 bound directly to DDX3X (Figure 1C, pink). However, when DDX3X K230E was incubated in the presence of 100 μM RK-33 this overlapping species was absent (Figure 1D, pink), indicating a lack of complex formation. These findings imply that RK-33 binds residue K230 within the Walker A motif, which forms part of the DDX3X active site.

The thermodynamics of RK-33 binding to DDX3X was measured by ITC (Figure 1E,F). RK-33 bound weakly (Figure 1E), with only low c value hyperbolic curves [33] obtained when using higher concentrations of RK-33 (Figure 1F). Fitting to a one-site binding model enabled estimation of the dissociation constant (*K*_d_) of 33 μM.

### 3.2. RK-33 Inhibits DDX3X Catalytic Activities

**Figure 2:**
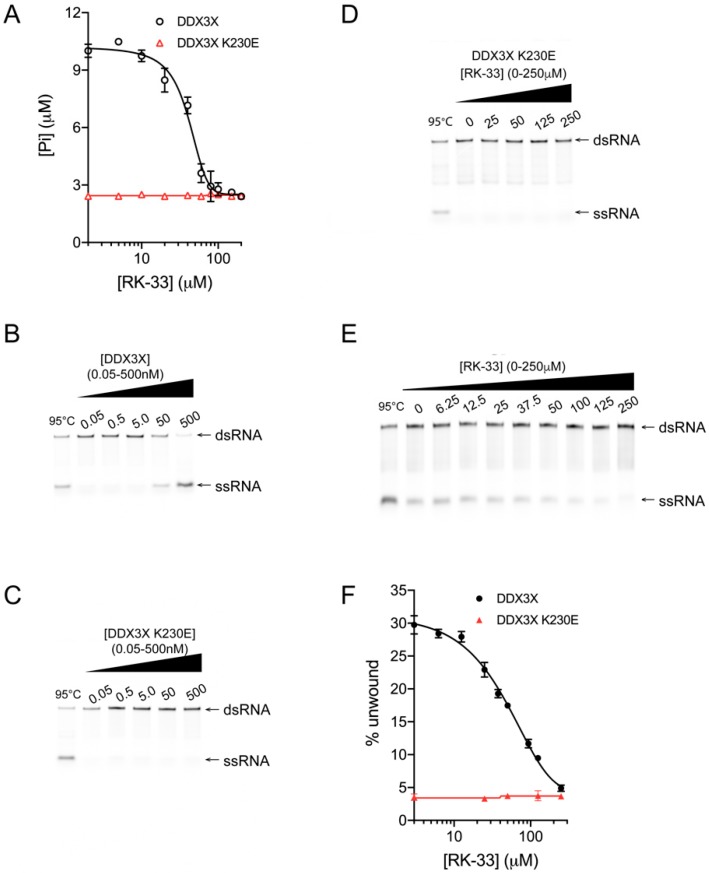
RK-33 inhibits DDX3X helicase and ATPase activity. DDX3X (black) and DDX3X K230E (red) proteins were assessed for their ability to (**A**) hydrolyze ATP and (**B**–**F**) unwind a synthetic analogue of duplex RNA (poly(I:C)), in the presence of increasing concentrations of RK-33 or equivalent % DMSO. (**A**) The effect of up to 200 μM RK-33 on the formation of free inorganic phosphate (Pi) by DDX3X (IC_50_ = 40 μM) was determined by a colorimetric assay, where free phosphate reacts with the Biomol^®^ Green reagent to initiate color development quantified at 620 nm. Results represent the mean +/− standard error of the mean (SEM) for pentaplicate wells from a single assay, representative of two (n = 2) independent experiments; (**B**,**C**) The capacity of DDX3X and DDX3X K230E to unwind Cy5-labeled double-stranded RNA (dsRNA) into single-stranded RNA (ssRNA) was determined by a helicase assay. Following incubation of 0–0.5 μM DDX3X or DDX3X K230E with dsRNA for 30 min at 37 °C, reactions were stopped by the addition of STOP solution (0.6% SDS, 60 mM EDTA, 40% (*w*/*v*) sucrose and 0.25% bromophenol blue). Cy5-labeled dsRNA and ssRNA were visualized by fluorescence on polyacrylamide gel. (**D**–**F**) The effect of RK-33 or equivalent % DMSO on the capacity of DDX3X to unwind dsRNA (IC_50_ = 35 μM) was determined. Cy5-labeled dsRNA and ssRNA were visualized by fluorescence on polyacrylamide gels in (**D**) and (**E**) were quantified using Image Studio Lite, with the ratio of the intensities for the ssRNA:dsRNA bands plotted as a % unwound in (**F**). Results in (**F**) represent the mean ± SEM for three independent experiments (n = 3)—of which, (**D**) and (**E**) are representative. A non-linear, sigmoidal dose response model was fit to the RK-33 concentration-activity data using GraphPad Prism 8 to generate figures. Lane 1 in each gel depicts heat-denatured RNA.

To assess the effect of RK-33 binding on DDX3X, increasing concentrations of the agent were added to a colorimetric assay detecting DDX3X’s ability to hydrolyze ATP and generate free Pi in the presence of poly(I:C). Inhibition was clearly evident, with a half maximal inhibitory concentration (IC_50_) of ~40 μM RK-33 (Figure 2A, black); the extent of maximal inhibition of DDX3X corresponded to the low levels of activity of the catalytically inactive DDX3X K230E mutant control [6] in either the absence or presence of RK-33 (Figure 2A, red). 

Next, we tested whether RK-33 could inhibit DDX3X from unwinding a double-stranded RNA (dsRNA) using a helicase unwinding assay, where the fluorescent RNA products are analyzed by quantitative polyacrylamide gel electrophoresis (PAGE). Purified recombinant DDX3X (Figure 1A) was incubated with an RNA duplex, in which one strand bears a 5′ Cy5 fluorescent label, in the absence or presence of increasing concentrations of RK-33. In the absence of RK-33, DDX3X (Figure 2B) but not the DDX3X K230E control (Figure 2C), was capable of unwinding the RNA duplex, as indicated by the appearance of ssRNA. As expected, DDX3X K230E remained incapable of unwinding RNA in the presence of RK-33 (Figure 2D, quantified in Figure 2F). In contrast, DDX3X unwinding activity was progressively impaired by increasing concentrations of RK-33, with an IC_50_ of 35 μM (Figure 2E, quantified in Figure 2F) aligning well with the results from the ATPase assay (Figure 2A). Together, these findings establish that RK-33 binding can inhibit DDX3X’s ability to both hydrolyze ATP and unwind RNA, consistent with RK-33 binding to the Walker A motif.

### 3.3. RK-33 Is a Potent Broad-Spectrum Antiviral Agent

**Figure 3:**
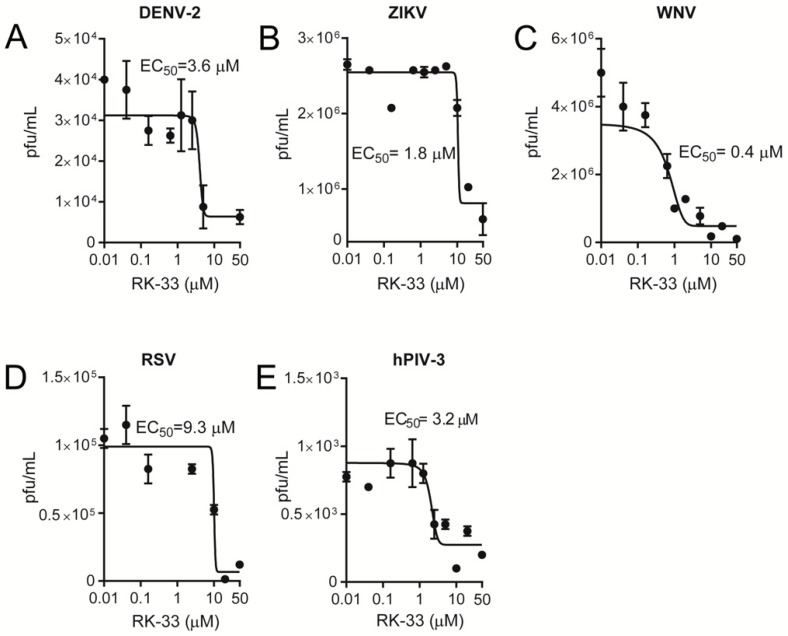
RK-33 is a broad-spectrum inhibitor of infectious virus. Vero cells were infected with (**A**) dengue virus serotype 2 (DENV-2), (**B**) Zika virus (ZIKV), (**C**) West Nile virus (WNV), (**D**) respiratory syncytial virus (RSV) or (**E**) human parainfluenza virus type 3 (hPIV-3) at a multiplicity of infection (MOI) of 1 for 2 h, after which virus was removed, and fresh medium containing 2% FBS was supplemented with the indicated concentration of RK-33. Released virus samples (for hPIV-3, DENV-2, ZIKV and WNV) or cell-associated samples (for RSV) were collected 22 h later, and viral titres (expressed as plaque forming units (pfu) per mL) determined by plaque assay. Dose-response curves were plotted from the viral titres (pfu) versus the logarithmic value of the concentrations of RK-33. Results represent the mean +/− standard deviation (SD) for duplicate wells from a single assay, representative of two (n = 2) independent experiments. See Table 1 for pooled data from the two assays.

**Figure 4:**
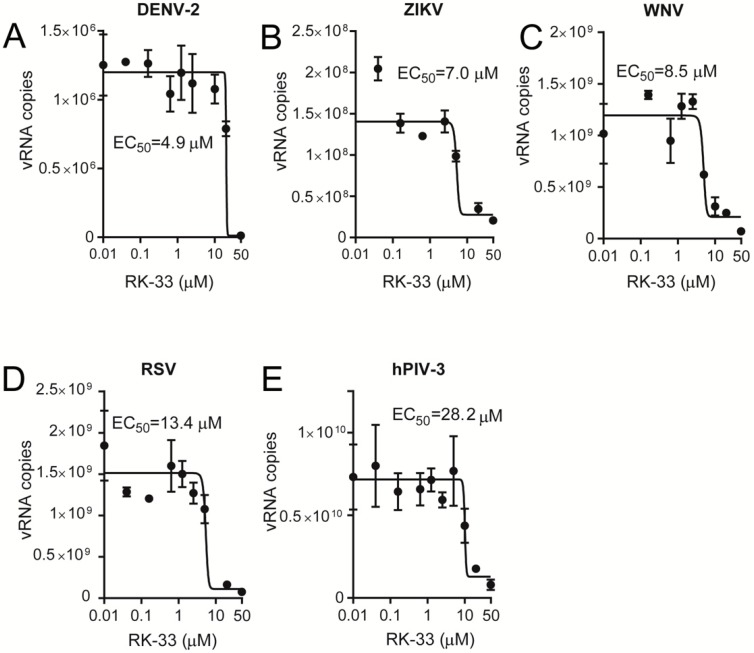
RK-33 is a broad-spectrum inhibitor of virus replication. Vero cells were infected with (**A**) DENV-2, (**B**) ZIKV, (**C**) WNV, (**D**) RSV or (**E**) hPIV-3 at an MOI of 1 for 2 h, after which virus was removed, and fresh medium containing 2% FBS or 2% FBS supplemented with the indicated concentration of RK-33. Released virus samples (for hPIV-3, DENV-2, ZIKV and WNV) or cell-associated samples (for RSV) were collected 22 h later, and viral RNA (vRNA) copies determined by qRT-PCR. Dose-response curves were plotted from the vRNA copies versus the logarithmic value of the concentrations of RK-33. Results represent the mean +/− SD for duplicate wells from a single assay, representative of two (n = 2) independent experiments. See Table 1 for pooled data from the two assays.

Since RK-33 has only been tested for its anticancer properties, we tested its ability to limit infection by key members of the *Paramyxoviridae* (RSV and hPIV-3) and *Flaviviridae* (WNV, DENV-2, ZIKV) families. Vero cells were infected at an MOI of 1 with RSV [34], hPIV-3, DENV-2 [30,35], ZIKV [32,35] or WNV [35], followed 2 h later by the addition of increasing concentrations of RK-33. Then, 22 h later, virus production was quantified by plaque assays and qRT-PCR analysis of the cell supernatant (DENV-2, ZIKV, WNV, hPIV-3) and cell lysates (RSV). Strikingly, RK-33 potently inhibited not only DENV-2, ZIKV and WNV (Figure 3A–C) but also RSV and hPIV-3 replication (Figure 3D,E), with EC_50_s of ≤10 μM (see Table 1 for pooled data). These results were consistent with qRT-PCR analysis indicating that RK-33 inhibited all viruses with comparable EC_50_ values (Figure 4, Table 1).

Importantly, the viral inhibitory effects of RK-33 were not due to cytotoxic effects, as indicated by XTT cell viability assays, which revealed that RK-33 concentrations below 30 μM had no impact on Vero cell numbers/viability (Supplementary Figure S1).

## 4. Discussion

Even though viral infections pose a significant threat to human health, antivirals to combat them are few and often lead to the development of drug resistance. A strategy to avoid drug resistance is to develop antivirals targeting host cell co-factors critical to viral replication. DDX3X is a prime host target for multiple viruses of medical significance, being required for replication of HIV-1 [5,6] and flaviviruses such as Japanese encephalitis virus (JEV) [36] and HCV [7,8]. 

RK-33 is a REN, containing the imidazo[4,5-e][1,3]diazepine ring system that is known for its anticancer properties [9,11,15,16]. RK-33 has been suggested to target DDX3X based on coimmunoprecipitation studies and work examining DDX3X’s yeast orthologue Ded1p (~53% sequence identity) [11]. Here, using AUC and ITC, we establish for the first time that RK-33 binds directly to DDX3X at low μM concentrations, likely within the Walker A motif that contributes to ATP-binding. Consistently, we also confirm using an ATPase assay and fluorescence-based RNA unwinding assay that RK-33 inhibits DDX3X’s ATPase and RNA unwinding activities at μM concentrations. Bol and colleagues [11] reported inhibition of helicase activity of yeast Ded1p at lower (nM) RK-33 concentrations. The basis of this difference in sensitivity is likely due to differences in the proteins themselves. The DDX3X used in this study lacked the C-terminal 82 residues that play a role in oligomerization, which is important for RNA duplex binding and DDX3X’s catalytic activity [28,37]. Further to this full-length DDX3X and Ded1p are only 53% identical, have different substrate preferences, and differ quantitatively in their RNA unwinding catalytic efficiency [38], meaning that the nature of the RNA substrate differs for the two enzymes. This is also consistent with other studies showing marked differences in the efficacy of compounds against the same enzyme from a range of bacterial species [38].

REN analogues that modulate the helicase activity of target viral or host helicases have been shown to have antiviral activity against HIV-1 [21], HCV [22], JEV [22], WNV [22,23] and hepatitis B [19,20]. Consistent with the antiviral properties of REN derivatives and of agents targeting DDX3X, our virus infection studies reveal here for the first time that the DDX3X-targeting REN derivative RK-33 also has antiviral properties. Notably, RK-33 exhibited broad-spectrum antiviral activity, inhibiting the replication of RSV from the *Paramyxoviridae* family, and the closely related flaviviruses DENV and WNV. Further to this, we are the first to show that RK-33 can also inhibit the replication of additional members of the *Paramyxoviridae* and *Flaviviridae* families, namely hPIV-3 and ZIKV, respectively. Our findings suggest a hitherto unrecognized role for DDX3X in hPIV-3 and ZIKV replication and may further expand the list of viruses for which anti-DDX3X agents may be effective and reinforce the antiviral properties of REN derivatives.

Collectively, our findings support the idea that DDX3X-targeting agents have potential as broad-spectrum antiviral agents, and that RK-33 is one such agent, although it should be stressed that demonstration of RK-33 selectivity for DDX3X in a cellular context remains the focus of further study in this laboratory. Our data further suggest that RK-33′s ability to block DDX3X’s ATPase and RNA unwinding activities is the basis of its broad-spectrum antiviral properties.

## Figures and Tables

**Table 1:** Pooled half maximal effective concentration (EC_50_) data from replicate plaque assays and RT-qPCRs.

EC_50_ (µM) ^1^					
Virus	hPIV-3	RSV	DENV-2	ZIKV	WNV
**Plaque assay**	5.1 +/− 2.7 (2)	9.2 +/− 0.1 (2)	3.7 +/− 0.1 (2)	2.4 +/− 0.8 (2)	0.6 +/− 0.3 (2)
**qRT-PCR**	18.5 +/− 13.8 (2)	11 +/− 3.4 (2)	8.5 +/− 5.1 (2)	7.0 (1)	8.5 (1)

^1^ Results represent the mean +/− standard deviation (SD) (n) from analysis as per Figure 3 and Figure 4.

## Supplementary Materials

The following are available online at https://www.mdpi.com/2073-4409/9/1/170/s1, Figure S1: RK-33 is not toxic to Vero cells at concentrations effective at inhibiting hPIV-3, RSV and flavivirus infections.
