# Supplementary material for: RK-33 Is a Broad-Spectrum Antiviral Agent That Targets DEAD-Box RNA Helicase DDX3X
Source: Cells. 2020 Jan 9;9(1):170. doi: 10.3390/cells9010170 (PMC7016805; doi:10.3390/cells9010170)
Supplement: Supplementary file 1 [file cells-09-00170-s001.pdf]

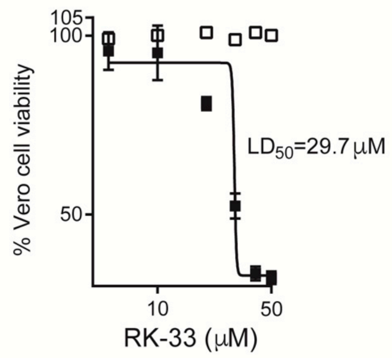

Supplementary Figure S1. RK-33 is not toxic to Vero cells at concentrations effective at inhibiting hPIV-3, RSV and flavivirus infections. Vero cell viability was determined by XTT (Sigma-Aldrich) assay with RK-33 (solid square symbol), or equivalent volume of the vehicle DMSO (open square symbol) treatment, as indicated in Materials and Methods. Cell survival is plotted relative to the DMSO control. Data are the mean +/- SD of quintuplicate wells from a representative assay from a series of two experiments.
